# Multifocal papillary thyroid microcarcinomas: is the total tumor diameter associated with the tumor behavior? A retrospective cohort study

**DOI:** 10.31744/einstein_journal/2025AO1724

**Published:** 2025-08-08

**Authors:** Larissa Ariane de Nardi, Ana Flávia Aguiar Ribeiro Coutinho, Carlos Segundo Paiva Soares, Simone Antunes Terra, Cristiano Claudino de Oliveira, Katia Hiromoto Koga, Sonia Marta Moriguchi, José Vicente Tagliarini, Gláucia Maria Ferreira da Silva Mazeto

**Affiliations:** 1 Internal Medicine Department Faculdade de Medicina de Botucatu Universidade Estadual Paulista “Júlio de Mesquita Filho” Botucatu SP Brazil Internal Medicine Department, Faculdade de Medicina de Botucatu, Universidade Estadual Paulista “Júlio de Mesquita Filho”, Botucatu, SP, Brazil.; 2 Ophthalmology, Otorhinolaryngology and Head and Neck Surgery Department Faculdade de Medicina de Botucatu Universidade Estadual Paulista “Júlio de Mesquita Filho” Botucatu SP Brazil Ophthalmology, Otorhinolaryngology and Head and Neck Surgery Department, Faculdade de Medicina de Botucatu, Universidade Estadual Paulista “Júlio de Mesquita Filho”, Botucatu, SP, Brazil.; 3 Pathology Department Faculdade de Medicina de Botucatu Universidade Estadual Paulista “Júlio de Mesquita Filho” Botucatu SP Brazil Pathology Department, Faculdade de Medicina de Botucatu, Universidade Estadual Paulista “Júlio de Mesquita Filho”, Botucatu, SP, Brazil.; 4 Department of Pathological Anatomy A.C.Camargo Cancer Center São Paulo SP Brazil Department of Pathological Anatomy, A.C.Camargo Cancer Center, São Paulo, SP, Brazil.; 5 Nuclear Medicine Department Faculdade de Medicina de Botucatu Universidade Estadual Paulista “Júlio de Mesquita Filho” Botucatu SP Brazil Nuclear Medicine Department, Faculdade de Medicina de Botucatu, Universidade Estadual Paulista “Júlio de Mesquita Filho”, Botucatu, SP, Brazil.

**Keywords:** Papillary thyroid microcarcinoma, Lymphatic metastasis, Neoplastic processes, Lymph nodes, Lymph node excision, Thyroglobulin

## Abstract

**Objective:**

To evaluate whether the total tumor diameter (sum of the largest diameters of all foci) predicts tumor aggressiveness at initial presentation and treatment response at 1-year follow-up in patients with multifocal papillary thyroid microcarcinoma.

**Methods:**

A retrospective analysis was conducted on 475 patients with differentiated thyroid carcinoma. Fifty-two patients with multifocal papillary thyroid microcarcinoma were included. The primary variable was total tumor diameter, and outcomes included initial tumor aggressiveness, evaluated according to the American Joint Committee on Cancer (AJCC; stages I or II), risk of recurrence according to the American Thyroid Association (low or not low), and response to therapy at 1 year (excellent or not).

**Results:**

AJCC stage II disease was significantly associated with age ≥55 years, lymph node dissection, angiolymphatic invasion, extrathyroidal extension, lymph node metastasis, number of metastatic lymph nodes, non-low risk of recurrence, first stimulated thyroglobulin, and response to therapy at 1-year. Non-low risk of recurrence were associated with Bethesda V/VI cytology, lymph node dissection, angiolymphatic and tumor capsule invasion, extrathyroidal extension, lymph node metastasis, number of metastatic lymph nodes, first stimulated thyroglobulin, and radioiodine dose. Non-excellent response at 1-year follow-up were associated with preoperative thyroid-stimulating hormone levels, lymph node metastasis, number of metastatic lymph nodes, and first stimulated thyroglobulin. Multivariate analysis revealed that only the number of metastatic lymph nodes independently predicted AJCC stage.

**Conclusion:**

Total tumor diameter was not associated with initial tumor aggressiveness or treatment response at 1-year follow-up in patients with multifocal papillary thyroid microcarcinoma.

## Highlights

■ In multifocal papillary thyroid microcarcinomas:

■ The total tumor diameter was not associated with aggressiveness or initial evolution.

■ Other factors are associated with disease outcomes.

■ The number of metastatic lymph nodes is an independent predictor of American Joint Committee on Cancer stage.

## In Brief

This study demonstrated that in multifocal papillary thyroid microcarcinomas, the sum of the largest diameters of all tumor foci was not associated with tumor aggressiveness or response to therapy at 1-year follow-up. Conversely, the total number of metastatic lymph nodes was an independent predictor of American Joint Committee on Cancer staging.

## Graphical abstract

**Figure:**
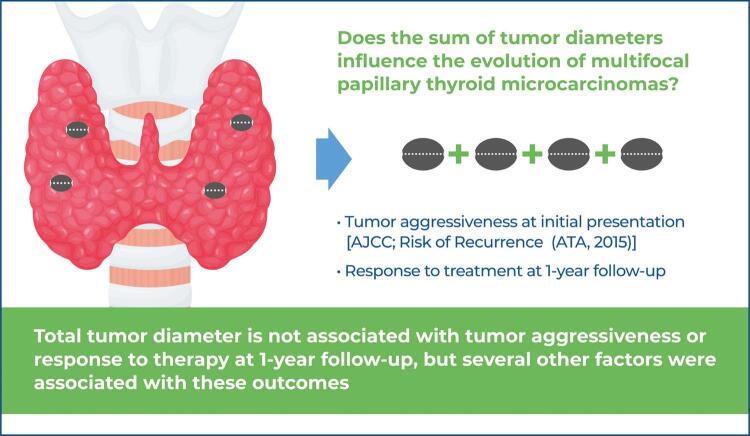

## INTRODUCTION

In recent decades, papillary thyroid microcarcinomas (PTMC) have been considered the main cause of increased thyroid cancer incidence.^(1)^ Their small dimensions, with diameters ≤1cm, and questionable aggressiveness suggest that their greater frequency may be due to the evolution of diagnostic method sensitivity.^(2)^

Although most PTMC are slow-growing tumors with a low recurrence risk and good 10-year survival,^(1,2)^ some tumors can display aggressive clinical behavior, with regional or distant metastasis and structural recurrence.^(2)^ This has prompted a search for potential predictors of aggressive disease, such as multifocality and tumor dimensions.

Papillary carcinomas often have multiple foci dispersed throughout the glandular parenchyma, resulting from the presence of multiple independent primary tumors or intraglandular dissemination of the primary tumor.^(3)^ Despite being associated with worse outcomes and appearing more frequently in lesions <1cm,^(4-8)^the latest guidelines do not consider multifocality as a poor prognostic factor in PTMC.^(9-11)^

Although tumor size is associated with prognosis,^(8,12,13)^the evaluation of this parameter in multifocal tumors is limited because only the largest lesion diameter is considered, ignoring the number of foci or the diameter of other lesions.^(12)^ Consequently, the risk associated with multifocal PTMC may be underestimated.^(14)^ To address this, some authors have proposed using the sum of the largest diameters of each tumor focus, referred to as the total tumor diameter (TTD), as a more reliable prognostic factor for multifocal PTMC, with TTD >1cm potentially indicating worse outcomes.^(12)^ However, the prognostic significance of TTD remains controversial, with differing results in the literature.^(5,11,14,15)^

### OBJECTIVE

This study aimed to evaluate whether total tumor diameter in patients with multifocal papillary thyroid microcarcinomas is associated with initial tumor aggressiveness and treatment response at 1-year follow-up.

## METHODS

This study was reported following the Strengthening the Reporting of Observational Studies in Epidemiology (STROBE) guidelines^(16)^ and was approved by the Research Ethics Committee of *Faculdade de Medicina de Botucatu, Universidade Estadual Paulista “Júlio de Mesquita Filho”* (CAAE: 30366020.9.0000.5411; #4.010.339).

### Study design

A retrospective cohort study was conducted by analyzing the attendance records of patients diagnosed with multifocal PTMC who underwent thyroidectomy at the Thyroid Neoplasm Outpatient Clinic of *Hospital das Clínicas de Botucatu* (HC-FMB), a tertiary hospital specializing in the treatment of thyroid cancer. The primary variable was TTD, and the main outcomes were initial tumor aggressiveness and treatment response at 1-year follow-up.

### Patient selection

**Figure 1:**
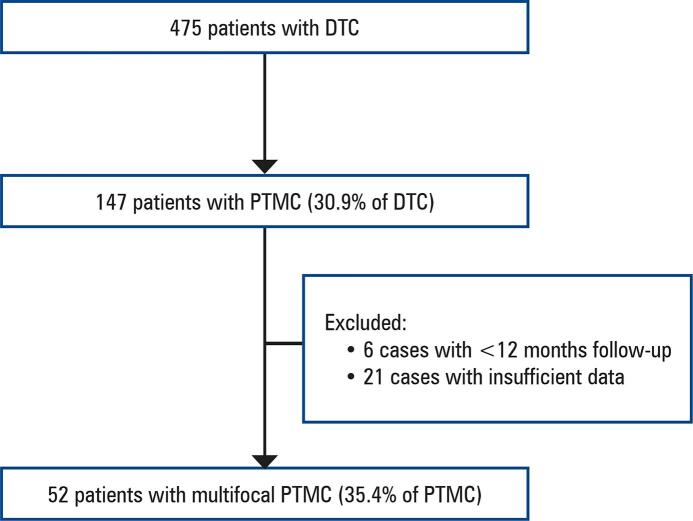
Patient selection process DTC: differentiated thyroid cancer; PTMC: papillary thyroid microcarcinomas.

Using convenience sampling, 475 records of patients with differentiated thyroid carcinomas were assessed. Of the total, 147 (30.9%) had PTMC, and 52 (35.4%) of these patients exhibited multifocal PTMC meeting the inclusion criteria (Figure 1).

### Inclusion and exclusion criteria

Patients with multifocal PTMC who underwent total thyroidectomy (TT) before January 2019, had at least 12 months of follow-up, and had sufficient clinical and histopathological data were included in the study. Cases that did not meet these criteria or those that presented with tumor foci with different anatomopathological diagnoses were excluded.

### Treatment and follow-up protocol

The standard treatment and follow-up protocol for patients with differentiated thyroid carcinomas in the HC-FMB service at the time of data collection consisted of TT, neck dissection when indicated by clinical, cytological, or ultrasound evidence of metastasis, followed by levothyroxine treatment, measurement of serum thyroglobulin (Tg), thyroid-stimulating hormone (TSH), and anti-Tg antibody (TgAb) levels, and cervical ultrasound (US) approximately 3 months after surgery. If the results of these tests were doubtful, a diagnostic whole-body scan (DWBS) with radioiodine (^131^I) and stimulated Tg (STg) after endogenous TSH stimulation (TSH >50 mIU/L) was performed.

After these procedures, routine treatment consisted of ablative/therapeutic dose of ^131^I with DWBS approximately 5 days later. Suspending levothyroxine for approximately 30 days before performing these procedures was recommended, with gradual reintroduction. DWBS was performed using a Gammatome T-9000 system comprised of an ACTi 3420 T gamma camera coupled to an IMAC computer and a pinhole collimator (CGR, France). Doses of ^131^I were administered by the Nuclear Medicine team of *Hospital Santa Casa de São Paulo* and, from December 2014, by the Nuclear Medicine Service at HC-FMB.

Treatment was individualized according to the initial extent of the disease, with doses around 30-100 mCi (3.70 GBq) ^131^I for low-risk cases; 150 mCi (5.55 GBq) for intermediate-risk cases; and 200-250 mCi (7.40GBq) for high-risk cases (T4 and M1 disease).

A follow-up DWBS, along with measurements of serum Tg, TgAb, TSH, and free thyroxine, and neck US, was conducted 1 year after initial therapy. Additional imaging, such as chest radiography, computed tomography, magnetic resonance imaging, or biopsy were requested if recurrence was suspected.

### Evaluated data

Papillary thyroid microcarcinomas was defined as papillary thyroid carcinoma ≤1cm in maximum diameter. Multifocality was defined as ≥2 foci of the same histological subtype.^(11)^TTD was defined as the sum of the largest diameters of all tumor foci.^(11)^

The outcomes were initial tumor aggressiveness and treatment response at 1-year follow-up after TT. Initial tumor aggressiveness was evaluated by staging cases in the postoperative period to determine the risk of disease recurrence and death. Disease recurrence was evaluated according to the American Thyroid Association (ATA) recommendations (low, intermediate, and high).^(9)^ The risk of mortality was assessed using the American Joint Committee on Cancer (AJCC) staging system (stages I-IVB).^(12)^Response to therapy 1 year after the initial treatment was assessed using the ATA dynamic rating system.^(9)^ Using Tg and TgAb measurement results in conjunction with imaging tests, specifically cervical US, patients were classified into excellent, indeterminate, biochemically incomplete, and structurally incomplete treatment responses. Each of these responses has been associated with patient evolution, with different percentages of disease recurrence or persistence during follow-up.^(9)^

Additional variables were evaluated, including sex, self-reported race, age at diagnosis, presence of preoperative compressive complaints (dysphagia, dyspnea, and dysphonia), TSH levels (reference value [RV]: 0.35-4.94mIU/L), and antithyroperoxidase antibody (RV<35.0 IU/L) concentrations. TSH levels were classified as hypothyroid (TSH >4.94mIU/L) or hyperthyroid (TSH <0.35 mIU/L). STg (analytical sensitivity: 0.2ng/mL; functional sensitivity for levels >2ng/mL: 0.9ng/mL) and TgAb (RV<4.11 IU/L) measurements, taken approximately 1 month after TT, were also evaluated. Laboratory tests were performed using chemiluminescence (DPC; Los Angeles, CA, USA). Preoperative US findings included the number of tumors and maximum diameters (mm). Preoperative cytological diagnoses were evaluated according to the Bethesda System (classes I, II, III, IV, or VI).^(17)^Images from US and cytological reports were reviewed to ensure that the preoperative cytological diagnosis corresponded to the fine-needle aspiration biopsies of the PTMC.

The following therapeutic data were evaluated: cervical lymph node dissection (yes or no) and ablative/therapeutic ^131^I (yes or no; mCi dose). Histological subtype (classic, follicular, or other variants) was recorded.^(18)^ The tumors were histologically evaluated for maximum diameter (mm), number of lesions, bilaterality, presence of tumor capsular and angiolymphatic invasion, extrathyroidal extension, cervical lymph node metastases, and diffuse lymphocytic thyroiditis. Imaging (DWBS, neck US, chest radiography, and tomography) and biopsy results were analyzed to assess the presence of an incomplete structural response to the initial therapy.

### Statistical analysis

Descriptive statistics were used to summarize qualitative (frequencies and percentages) and quantitative (mean, median, standard deviation [SD], and range) variables. Normality was assessed to verify the data distribution. Verification of outcome associations in relation to the categorized explanatory variables was performed using the χ^2^ or Fisher’s Exact Test, when necessary. Student’s *t-*test was used for continuous variables with normal distribution, otherwise a Generalized Linear Model with gamma or Poisson distribution (when counting variables) was applied. Univariate logistic regression was performed was performed to assess the effect of individual predictors. Variables with p<0.05 were included in the multivariate logistic regression model. Analyses were performed using SAS version 9.4. Statistical significance was set at p<0.05.

## RESULTS

Most patients were female (n=43; 82.7%) and self-identified as white (n=49; 96%), with a mean ± SD age of 48.79±14.87 years. Forty-seven (92.2%) and four (7.8%) patients were classified as AJCC stages I and II, respectively. There were no stage III or IV cases. Risk of recurrence was low in 36 patients (72%), intermediate in 10 (20%), and high in 4 (8%). Fifty patients were administered a therapeutic dose of ^131^I. One year after the initial treatment, 23 patients (46.9%) showed an excellent response, 13 (26.5%) showed an indeterminate response, 9 (18.4%) showed a biochemical incomplete response, and 9 (18.4%) showed a structural incomplete response.

With regard to initial tumor aggressiveness, comparing AJCC stages I and II (Table 1), we observed that stage I was significantly associated with age <55 years (36.2% *versus* 100%; p=0.0128), absence of lymph node dissection (39.5% *versus* 100.0%; p=0.02) and angiolymphatic invasion (7.9% *versus* 50%; p=0.0134), minimal extrathyroidal extension (2.2% *versus* 33.3%; p=0.009) and lymph node metastasis (14.9% *versus* 100.0%; p<0.0001), fewer metastatic lymph nodes (0.67±2.49 *versus* 11.00±5.10; p=0.0488), low risk of recurrence (78.3% *versus* 0.0%; p=0.0008), lower first STg (7.88±23.31 *versus* 60.83±94.26 ng/mL; p=0.0003), and lower frequency of incomplete response to therapy at 1 year (6.7% *versus* 50.0%; p=0.0305). Univariate analysis showed that the number of metastatic lymph nodes (p=0.0025) and first STg (p=0.0399) were associated with a lower likelihood of having stage I disease (Table 2). Multivariate analysis showed that only the number of metastatic lymph nodes remained a significant independent factor (odds ratio=0.565; 95% confidence interval: 0.353-0.904; p=0.0172).

**Table 1:** Association of the variables with tumor stage and risk of recurrence

Variables	Tumor stage		p value	Risk of recurrence		p value
	I	II		Low	Non-low	
Women^†^	40 (85.1)	2 (50)	0.0770	31 (86.1)	10 (71.4)	0.2250
Race (White)^†^	45 (95.7)	4 (100)	0.6738	34 (94.4)	14 (100)	0.3681
Age (years)^§^	47.55±14.85	61.75±10.84	0.0686	48.58±14.38	48.86±17.56	0.9549
≥55 years^†^	17 (36.2)	4 (100)	0.0128*	15 (41.7)	6 (42.9)	0.9390
Compression complaint^†^	10 (35.7)	0 (0)	0.2085	8 (40)	2 (20)	0.2733
Hypothyroidism^†^	13 (54.2)	2 (50)	0.8771	10 (58,8)	6 (50)	0.6559
Hyperthyroidism^†^	4 (16.7)	0 (0)	0.3778	4 (23,5)	0 (0)	0.0965
Preoperative TSH (ng/mL)^§^	2.31±3.17	1.49±0.37	0.3906	2.58±3.54	1.42±0.59	0.0739
Positive preoperative TPOAb^†^	7 (29.2)	1 (100)	0.1368	5 (25)	3 (60)	0,1335
US findings						
Number of tumor nodes^§^	2.38±1.82	2.00±0.00	0.6833	2.38±2.04	2.40±0.97	0.9744
Maximum diameter^§^	14.65±11.64	15.25±10.24	0.9113	16.15±13.35	12.50±5.90	0.3141
≥5 mm^†^	31(93.9)	4(100)	0.6127	23 (95.8)	12 (100)	0.4733
Cytological diagnosis (Bethesda)^†^			0.1968			0.0005*
I/II	6 (17.7)	0 (0)		6 (25)	0 (0)	
III/IV	10 (29.4)	1 (50)		10 (41,7)	0 (0)	
V/VI	18 (52.9)	1 (50)		8 (33.3)	13 (100)	
Lymph node dissection^†^	17 (39.5)	4 (100)	0.02*	9 (28.1)	12 (85.7)	0.0003*
Histological subtypes of PTC^†^			0.8618			0.5151
Classic	30 (63.8)	3 (75.0)		22 (61.1)	10 (71.4)	
Follicular	15 (31.9)	1 (25.0)		13 (36.1)	3 (21.4)	
Others	2 (4.3)	0 (0.0)		1 (2.8)	1 (7.1)	
Anatomopathological						
Largest tumor diameter (mm)^§^	6.39±2.47	8.00±2.45	0.2175	6.17±2.63	7.43±1.99	0.1139
Bilateral^†^	32 (72.7)	2 (66.7)	0.8204	24 (72.7)	9 (69.2)	0.8125
Number of tumors^§^	3.32±1.70	3.00± 1.41	0.8088	3.35± 1.55	3.27±2.10	0.8992
TTD^§^	12.32±5.68	13.50±6.36	0.7781	12.44± 5.85	12.40±5.66	0.9843
TTD >10 mm^†^	18 (56.3)	1 (50)	0.8629	13 (56.5)	5 (50)	0.7295
Angiolymphatic invasion^†^	3 (7.9)	2 (50)	0.0134*	0 (0)	5 (41.7)	0.0002*
Tumor capsule invasion^†^	7 (30.4)	1 (33.3)	0.9185	2 (13.3)	6 (60)	0.0143*
Extrathyroid extension^†^	1 (2.2)	1 (33.3)	0.009*	0 (0)	2 (16.7)	0.0136*
Lymphocytic thyroiditis/Hashimoto^†^	12 (31.6)	1 (33.3)	0.9499	10 (33.3)	3 (30)	0.8455
Lymph Node Metastasis ^†^	7 (14.9)	4 (100)	<0.0001*	2 (5.7)	9 (64.3)	<0.0001*
Central	5 (11.1)	3 (100)	<0.0001*	3 (5.7)	6 (50)	0.0004*
Lateral	3 (6.7)	3 (100)	<0.0001*	0 (0)	6 (50)	<0.0001*
Number of lymph node metastasis^§^	0.67±2.49	11.00±5.10	0.0488*	0.20±0.83	4.86±6.24	<0.0001*
Low recurrence risk^†^	36 (78.3)	0 (0)	0.0008*	-	-	-
First STg (ng/mL)^§^	7.88±23.31	60.83±94.26	0.0003*	3.74±5.88	35.59±64.87	<0.0001*
^131^I (mCi dose)^§^	157.48±112.98	206±41.33	0.2613	140.14±62.31	214.62 ±180.59	0.0009*
Response to therapy at 1-year^†^			0.0305*			0.5169
Excellent	23 (51.1)	0 (0)		18 (51.4)	5 (38.5)	
Indeterminate	12 (26.7)	1 (25)		10 (28.6)	3 (23.1)	
Biochemical incomplete	3 (6.7)	1 (25)		2 (5.7)	1 (7.7)	
Structural incomplete	4 (8.9)	0 (0)		3 (8.6)	1 (7.7)	
Biochemical or structural incomplete	3 (6.7)	2 (50)		2 (5.7)	3 (23.1)	

^†^n (%); ^§^mean ± standard deviation; *p<0.05.

^131^I: radioiodine; mm: millimeters; mCi: milliCurie; ng/mL: nanograms per milliliter; PTC: papillary thyroid carcinoma; STg: stimulated thyroglobulin; TPOAb: anti-thyroperoxidase antibody; TTD: total tumor diameter.

**Table 2:** Univariate logistic regression analysis for stage I disease and low risk of recurrence

Variables	Stage I				Low risk			
	Estimate	OR	95% CI	p value	Estimate	OR	95% CI	p value
Age	-0.0964	0.908	(0.814-1.013)	0.0830	-0.00122	0.999	(0.958-1.041)	0.9539
Preoperative TSH	0.2603	1.297	(0.469-3.590)	0.6162	0.4308	1.538	(0.690-3.429)	0.2922
Preoperative T4L	12.531	3.501	(0.048-256.012)	0.5672	0.8982	2.455	(0.157-38.452)	0.5222
Preoperative TPOAb	0.000686	1.001	(0.972-1.030)	0.9625	0.000961	1.001	(0.987-1.015)	0.8929
Number of tumor nodes at US	0.1627	1.177	(0.490-2.828)	0.7162	-0.00646	0.994	(0.642-1.538)	0.9769
Maximum diameter at US	-0.00457	0.995	(0.911-1.088)	0.9197	0.0346	1.035	(0.959-1.118)	0.3758
Larger tumor diameter	-0.3114	0.732	(0.441-1.216)	0.2287	-0.22	0.802	(0.609-1.057)	0.1174
Number of tumors at AP	0.1384	1.148	(0.412-3.203)	0.7914	0.0297	1.030	(0.678-1.566)	0.8893
TTD at AP	-0.0363	0.964	(0.755-1.231)	0.7708	0.00138	1.001	(0.877-1.143)	0.9837
Number of LM	-0.3756	0.687	(0.539-0.876)	0.0025*	-0.727	0.483	(0.259-0.903)	0.0226*
Number of ipsilateral central LM	-0.6116	0.542	(0.146-2.012)	0.3604	-0.0376	0.963	(0.377-2.459)	0.9374
First STg	-0.0191	0.981	(0.963-0.999)	0.0399*	-0.0992	0.906	(0.824-0.995)	0.0398*
First PO TgAb	0.00176	1.002	(0.978-1.026)	0.8846	0.00325	1.003	(0.986-1.021)	0.7198
^131^I dose	-0.00244	0.998	(0.991-1.004)	0.4861	-0.00744	0.993	(0.983-1.003)	0.1488

The p-values were derived from univariate logistic regression models. *p<0.05.

AP: anatomopathological; 95%CI: 95% confidence interval; ^131^I: radioiodine; LM: lymph node metastasis; OR: odds ratio; PO: postoperative; STg: stimulated thyroglobulin; TgAb: antithyroglobulin antibody; TPOAb: antithyroperoxidase antibody; TTD: total tumor diameter; US: ultrasound.

Comparing low and non-low risk of recurrence (Table 1), we observed that low risk was associated with Bethesda I/II cytological classifications (25.0% *versus* 0.0%; p=0.0005), lower percentages of lymph node dissection (28.1% *versus* 85.7%; p=0.0003), absence of angiolymphatic invasion (0.0% *versus* 41.7%; p=0.0002) and tumor capsule invasion (13.3% *versus* 60%; p=0.0143), minimal extrathyroidal extension (0.0% *versus* 16.7%; p=0.0136) and lymph node metastasis (5.7% *versus* 64.3%; p<0.0001), and lower number of metastatic lymph nodes (0.20±0.83 *versus* 4.86±6.24; p<0.0001), first STg (3.74±5.88 *versus* 35.59±64.87 ng/mL; p<0.0001) and ^131^I dose (140.14±62.31 *versus* 214.62±180.59 mCi; p=0.0009). Univariate analysis showed that the number of metastatic lymph nodes (p=0.0226) and first STg (p=0.0398) were negatively associated with low recurrence risk (Table 2). Multivariate analysis did not identify any independent predictors.

As for the response to therapy at 1-year follow-up after the initial treatment, an excellent response was significantly associated with higher preoperative TSH levels (3.00±4.47 *versus* 1.65±0.85 ng/mL; p=0.0193), absence of lymph node metastasis (9.1% *versus* 33.3%; p=0.0431), and lower number of metastatic lymph nodes (0.24±0.89 *versus* 2.59±5.05; p=0.01) and first STg (3.60±5.15 *versus* 19.26±5.05 ng/mL; p=0.0115; Table 3). Univariate analysis showed that no single parameter influenced the outcome (Table 4); hence, multivariate analysis could not be performed. TTD was not associated with any of the evaluated outcomes.

**Table 3:** Association between clinical-epidemiological variables and excellent response at 1-year follow-up

	Excellent response		p value
	No 27 (51.9%)	Yes 22 (42.3%)	
Women^†^	20 (74.1)	20 (90.9)	0.1301
Race (White)^†^	25 (92.6)	22 (100)	0.1924
Age (years)^§^	49.11±13.92	50.05±15.61	0.8258
≥55 years^†^	10 (37)	11 (50)	0.3618
Compression complaint^†^	6 (33.3)	4 (33.3)	1
Hypothyroidism^†^	9 (56,25)	6 (50)	0.7428
Hyperthyroidism^†^	2 (12.5)	2 (16.7)	0.7552
Preoperative TSH (ng/mL)^§^	1.65±0.85	3.00±4.47	0.0193*
Positive preoperative TPOAb^†^	4 (30.8)	4 (33.3)	0.6210
US findings			
Number of tumor nodes^§^	2.11±1.32	2.64±2.17	0.3307
Maximum diameter^§^	15.93±13.52	12.71±6.41	0.3465
≥5 mm^†^	21 (91.3)	14 (100)	0.2566
Cytological diagnosis (Bethesda)^†^			0.2154
I/II	1 (5.6)	5 (26.3)	
III/IV	5 (27.8)	5 (26.3)	
V/VI	12 (66.7)	9 (47.4)	
Lymph node dissection^†^	11 (42.3)	9 (47.4)	0.7358
Histological subtypes of PTC^†^			0.4276
Classic	17 (63)	15 (68.2)	
Follicular	8 (29.6)	7 (31.8)	
Others	2 (7.4)	0 (0)	
Anatomopathological			
Largest tumor diameter (mm)^§^	6.40±2.24	6.53±2.82	0.8559
Multifocal^†^	27 (100)	22 (100)	
Bilateral^†^	21 (84)	12 (60)	0.0704
Number of tumors^§^	3.57±1.95	2.64± 1.37	0.3947
TTD (mm)^§^	12.11±5.10	13.44±6.75	0.5316
TTD >10mm^†^	11 (50)	8 (72.7)	0.2130
Angiolymphatic invasion^†^	4 (20)	1 (5)	0.1515
Tumor capsule invasion^†^	3 (21.4)	4 (36.4)	0.4090
Extrathyroidal extension^†^	1 (4.2)	1 (4.6)	0.9498
Lymphocytic thyroiditis/Hashimoto^†^	3 (30)	10 (33.3)	0.8455
Lymph node metastasis^†^	9 (33.3)	2 (9.1)	0.0431*
Central	7 (28)	1 (4.8)	0.0383*
Lateral	5 (20)	1 (4.8)	0.1264
Total number of LM^§^	2.59±5.05	0.24±0.89	0.01*
First STg (ng/mL)^§^	19.26±5.05	3.60±5.15	0.0115*
^131^I dose (mCi)^§^	179.38±144.77	142.05±35.83	0.0835
AJCC stage (I)^†^	23 (85.2)	22 (100)	0.0596
Recurrence risk (low)^†^	17 (65.4)	18 (81.8)	0.1471

^†^ n (%); ^§^ mean ± standard deviation; *p<0.05.

AJCC: American Joint Committee on Cancer; ^131^I: radioiodine; mm, millimeters; mCi: milliCurie; ng/mL: nanograms per milliliter; PTC: papillary thyroid carcinoma; STg: stimulated thyroglobulin; TPOAb: anti-thyroperoxidase antibody; TTD: total tumor diameter; LM: lymph node metastasis.

**Table 4:** Univariate logistic regression analysis for excellent response at 1 year

Variables	Estimate	OR	95%CI	p value
Age	0.00452	1.005	(0.966-1.045)	0.8214
Preoperative TSH	0.2153	1.240	(0.853-1.803)	0.2596
Preoperative TPOAB	-0.00265	0.997	(0.986-1.009)	0.6438
Number of tumor nodes at US	0.1869	1.206	(0.783-1.857)	0.3965
Maximum diameter at US	-0.0293	0.971	(0.906-1.041)	0.4089
Larger tumor diameter	0.0221	1.022	(0.810-1.290)	0.8521
Number of tumors at AP	-0.1748	0.840	(0.577-1.223)	0.3620
TTD at AP	0.0426	1.044	(0.917-1.188)	0.5192
Number of LM	-0.3883	0.678	(0.392-1.173)	0.1650
Number ipsilateral central LM	-0.0634	0.939	(0.427-2.065)	0.8748
First STg	-0.0632	0.939	(0.854-1.032)	0.1899
Postoperative TgAb	-0.0212	0.979	(0.906-1.058)	0.5899
^131^I dose	-0.00489	0.995	(0.985-1.005)	0.3278

The p-values were derived from univariate logistic regression models. *p<0.05.

AP: anatomopathological; 95%CI: 95% confidence interval; ^131^I: radioiodine; LM: lymph node metastasis; OR: odds ratio; STg: stimulated thyroglobulin; TgAb: antithyroglobulin antibody; TPOAb: antithyroperoxidase antibody; TTD: total tumor diameter; US: ultrasound.

## DISCUSSION

In this study of patients with multifocal PTMC, we observed that some clinical, therapeutic, laboratory, and histological factors, but not TTD, were associated with both initial tumor aggressiveness and treatment response at 1-year follow-up.

AJCC staging was used to evaluate initial tumor aggressiveness.^(12)^When comparing stages I and II, we found an association between the less advanced stage and parameters frequently associated with less aggressive conditions, such as age <55 years,^(6,12)^ absence of lymph node dissection, angiolymphatic invasion, extrathyroidal extension, and lymph node metastasis,^(12)^fewer metastatic lymph nodes, low risk of recurrence,^(9)^lower first STg, and lack of incomplete response at 1-year follow-up.^(19)^While many of these variables are components of the AJCC system, the univariate analysis revealed that only the number of metastatic lymph nodes and first STg remained as influencing factors for stage I disease, while the multivariate analysis showed that only the number of affected lymph nodes persisted as an independent predictor. It is important to emphasize that although the presence of lymph node metastasis is considered in AJCC staging, the number of affected lymph nodes is not accounted for in the staging criteria.^(12)^

Another parameter that was used to evaluate initial tumor aggressiveness was the risk of recurrence according to the ATA.^(9)^Low risk was also associated with factors often related to less tumor aggressiveness, such as lower percentage of lymph node dissection,^(20)^angiolymphatic invasion, tumor capsule invasion, extrathyroidal extension,^(7,11,14)^and lymph node metastasis.^(5,7,11,14)^The association between some of these parameters and this system is expected because several of them are integrated for this staging. A recent Brazilian study reported that age <55 years and lymph node metastasis were important predictors of disease recurrence.^(7)^However, we did not assess the risk of recurrence. Our study only evaluated multifocal PTMC, whereas the previous study evaluated PTMC in general and found that multifocality was an independent predictor of disease recurrence,^(7)^ which has already been reported in other studies.^(5,6)^A low risk of recurrence was also associated with fewer metastatic lymph nodes and lower concentrations of the first STg and ^131^I, confirming the reported association between STg and tumor aggressiveness.^(19,21,22)^Interestingly, our study found an association between low recurrence risk and Bethesda I/II cytological diagnoses. This contrasts with our previous study that included differentiated thyroid carcinomas in general, in which no such association was found.^(23)^Again, our study only evaluated multifocal PTMCs making study comparison difficult. Although the univariate analysis showed that the number of metastatic lymph nodes and the first STg remained were significant predictors of recurrence risk, none of the variables remained independently significant in the multivariate analysis.

Our study also revealed an association between an excellent response at 1-year follow-up and higher preoperative serum TSH levels, fewer lymph node metastases, and lower first STg, consistent with other studies. An association was observed between high TSH levels and thyroid cancer and advanced disease (extrathyroid extension).^(24,25)^ Postoperative serum Tg level can be used as an early marker of residual or metastatic differentiated thyroid carcinoma,^(19)^ and as a prognostic marker in papillary thyroid carcinoma.^(21)^ In a recent study, both Tg >10 ng/mL and lymph node metastasis were associated with an unfavorable treatment response at 12-18 months after the initial treatment.^(22)^With regard to PTMC, we observed the prognostic value of the first STg as a predictor of disease persistence and recurrence.^(26)^ However, in the previous study, only 37% of PTMC were multifocal, whereas all tumors in the present study were multifocal, making comparison difficult.

Studies have reported an association between TTD and initial tumor aggressiveness in multifocal PTMCs.^(3,5,11,14,15)^Total tumor diameter >1cm was associated with a higher risk of capsular invasion,^(15)^central lymph node metastasis,^(5,11,14)^extrathyroidal extension, and a worse prognosis.^(11,14)^However, previous studies compared multifocal and unifocal PTMC,^(4,5,11,14)^ which was not done in our study. Neither the largest tumor diameter, a factor often associated with tumor aggressiveness,^(6,13-15)^nor the TTD^(5,11,14)^showed an association with AJCC stage or risk of recurrence. No influence or association was observed between TTD and treatment response at 1-year follow-up. Zhou et al. similarly found no relationship between TTD and adverse tumor behavior.^(15)^

This study has some limitations, including its retrospective design, use of convenience sampling, relatively small sample size, therapeutic approach (TT/radioiodine), and that some of the studied variables were part of the outcome scales. Despite these limitations, the strengths of this study include the uniform treatment of all patients and exclusive focus on strictly defined multifocal PTMC, providing relevant information on factors associated with more aggressive behavior and worse response to therapy. Further studies addressing these limitations should be conducted to validate our findings.

## CONCLUSION

In conclusion, although total tumor diameter was not associated with initial tumor aggressiveness or treatment response at 1-year follow-up, we observed that several other factors were associated with these outcomes. Notably, the total number of metastatic lymph nodes persisted as an independent predictor of American Joint Committee on Cancer staging. These findings suggest that total tumor diameter may have limited prognostic value for patients with papillary thyroid microcarcinomas.
